# Supplementary material for: Identification of a BACH1 lung cancer signature: A novel tool for understanding BACH1 biology and identifying new inhibitors
Source: Redox Biol. 2025 Jul 23;85:103789. doi: 10.1016/j.redox.2025.103789 (PMC12314328; doi:10.1016/j.redox.2025.103789)
Supplement: Multimedia component 1 [file mmc1.docx]

**Identification of a BACH1 lung cancer signature: A novel tool for understanding BACH1 biology and identifying new inhibitors.**

Donika Klenja-Skudrinja, Kevin X. Ali, David Walker, Maureen Higgins, Angana AH Patel, Dorota Raj, Anna Creelman, Charlotte McDowall, Conor Taylor, Tomasz Wenta, Erik Larsson, Clotilde Wiel, Volkan I. Sayin, and Laureano de la Vega.

**Supplementary Material and Methods**

Taqman probes used:

List of reported BACH1 targets tested:
