# Supplementary material for: Identification of a BACH1 lung cancer signature: A novel tool for understanding BACH1 biology and identifying new inhibitors
Source: Redox Biol. 2025 Jul 23;85:103789. doi: 10.1016/j.redox.2025.103789 (PMC12314328; doi:10.1016/j.redox.2025.103789)
Supplement: Multimedia component 5 [file mmc5.pdf]

A

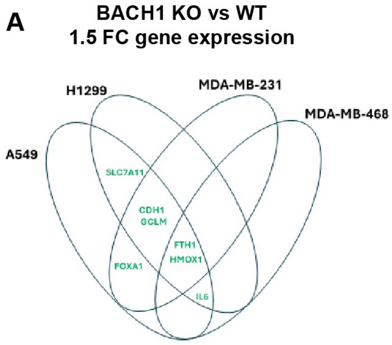

B

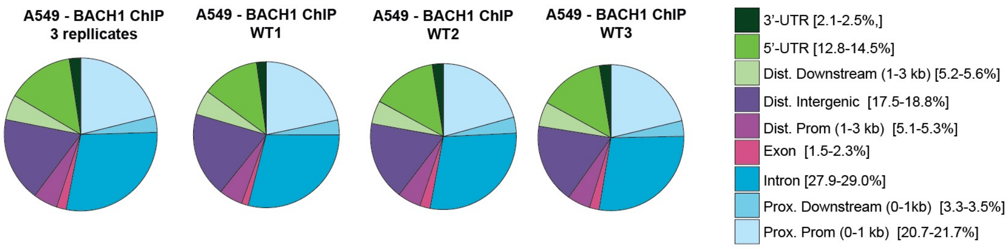

C Known motifs retrieved

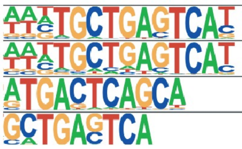

Name	P-value	log P-pvalue	q-value (Benjamini)	# Target Sequences with Motif	% of Targets Sequences with Motif	# Background Sequences with Motif	% of Background Sequences with Motif
Bach1(bZIP)/K562-Bach1-ChIP-Seq(GSE31477)/Homer	1e-14620	-3.367e+04	0.0000	7985.0	57.82%	157.5	0.46%
NFE2L2(bZIP)/HepG2-NFE2L2-ChIP-Seq(Encode)/Homer	1e-13684	-3.151e+04	0.0000	7354.0	53.25%	130.9	0.38%
NF-E2(bZIP)/K562-NFE2-ChIP-Seq(GSE31477)/Homer	1e-13383	-3.082e+04	0.0000	7658.0	55.45%	179.8	0.52%
Bach2(bZIP)/OCILy7-Bach2-ChIP-Seq(GSE44420)/Homer	1e-13348	-3.074e+04	0.0000	9732.0	70.47%	624.0	1.81%

D

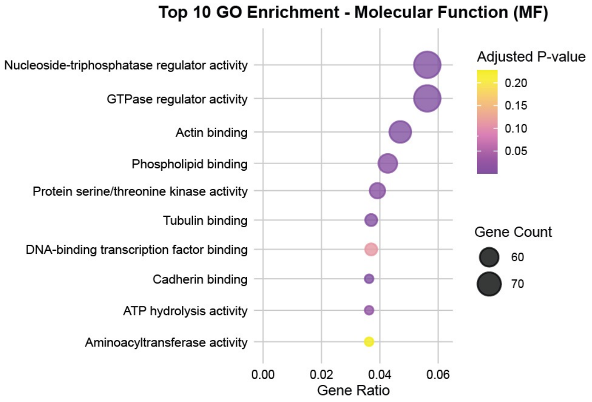

Suppl S1

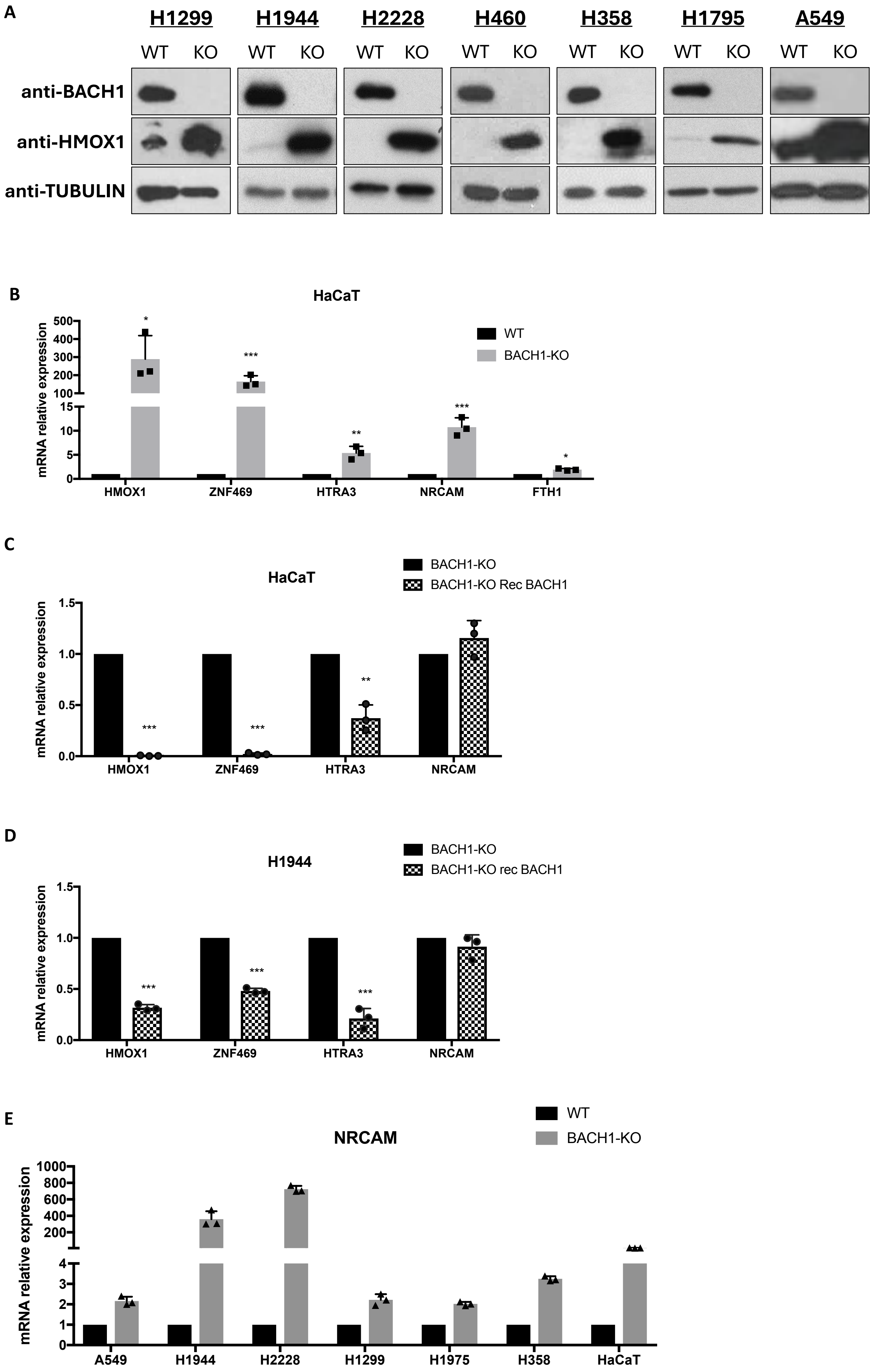
A
H1299
H1944
H2228
H460
H358
H1795
A549
WT
KO
anti-BACH1
anti-HMOX1
anti-TUBULIN
B
HaCaT
WT
BACH1-KO
mRNA relative expression
HMOX1
ZNF469
HTRA3
NRCAM
FTH1
C
HaCaT
BACH1-KO
BACH1-KO Rec BACH1
mRNA relative expression
HMOX1
ZNF469
HTRA3
NRCAM
D
H1944
BACH1-KO
BACH1-KO rec BACH1
mRNA relative expression
HMOX1
ZNF469
HTRA3
NRCAM
E
NRCAM
WT
BACH1-KO
mRNA relative expression
A549
H1944
H2228
H1299
H1975
H358
HaCaT

Suppl S2

F

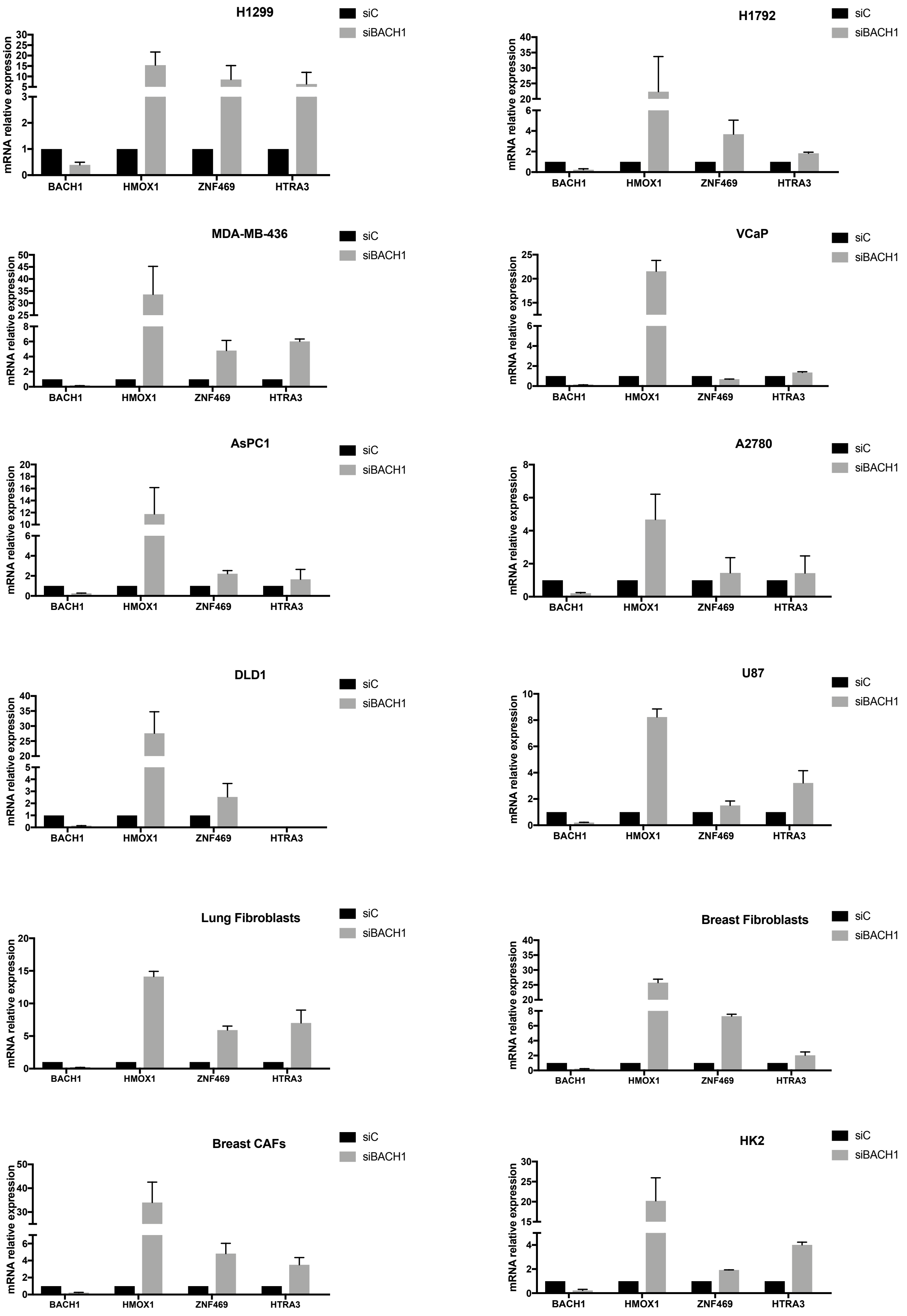
H1299
H1792
MDA-MB-436
VCaP
AsPC1
A2780
DLD1
U87
Lung Fibroblasts
Breast Fibroblasts
Breast CAFs
HK2
siC
siBACH1
mRNA relative expression
BACH1
HMOX1
ZNF469
HTRA3

Suppl S2

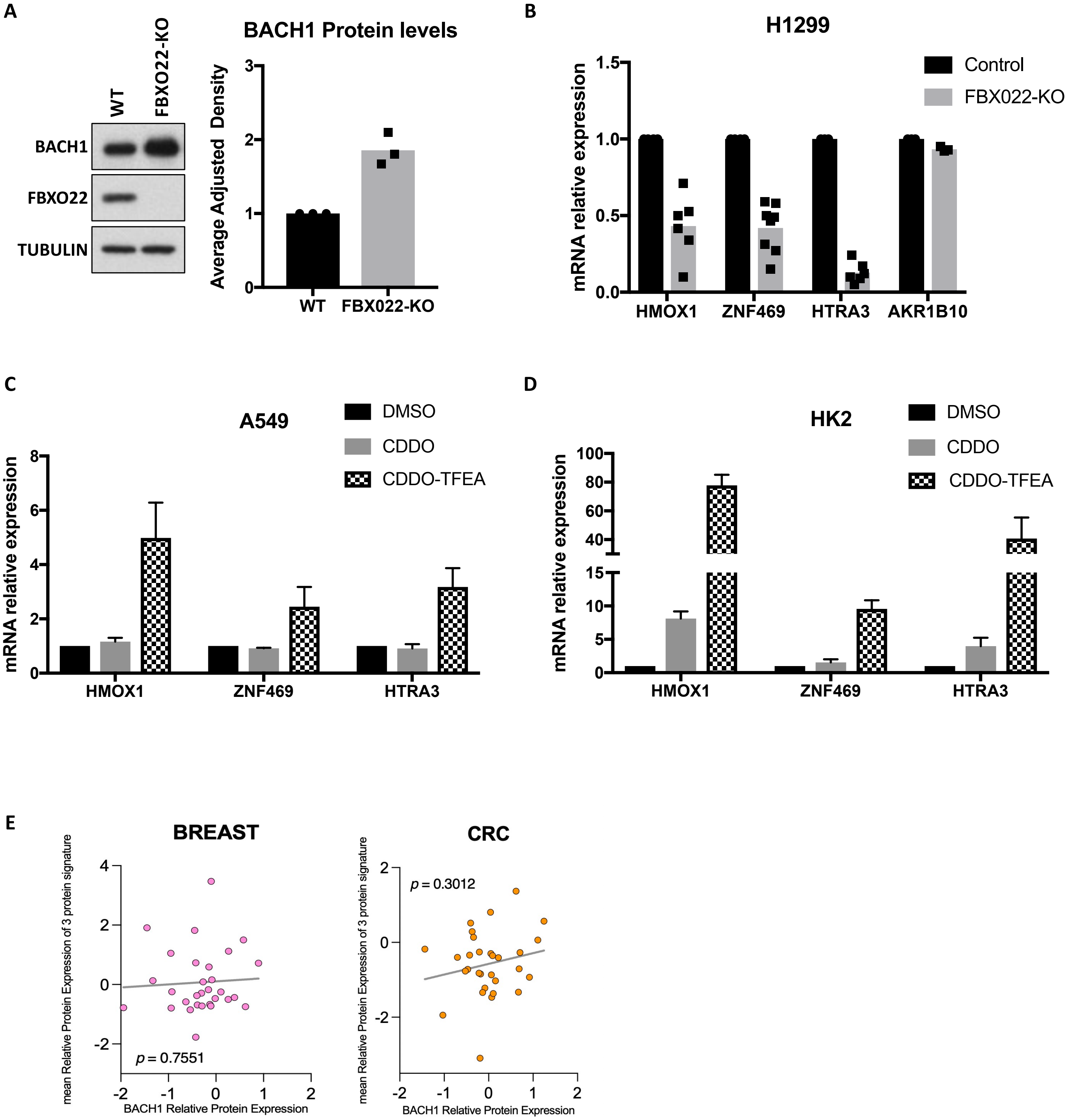
A
WT
FBXO22-KO
BACH1
FBXO22
TUBULIN
BACH1 Protein levels
Average Adjusted Density
WT
FBX022-KO
B
H1299
Control
FBX022-KO
mRNA relative expression
HMOX1
ZNF469
HTRA3
AKR1B10
C
A549
DMSO
CDDO
CDDO-TFEA
mRNA relative expression
HMOX1
ZNF469
HTRA3
D
HK2
DMSO
CDDO
CDDO-TFEA
mRNA relative expression
HMOX1
ZNF469
HTRA3
E
BREAST
mean Relative Protein Expression of 3 protein signature
p = 0.7551
BACH1 Relative Protein Expression
CRC
mean Relative Protein Expression of 3 protein signature
p = 0.3012
BACH1 Relative Protein Expression

Suppl S3

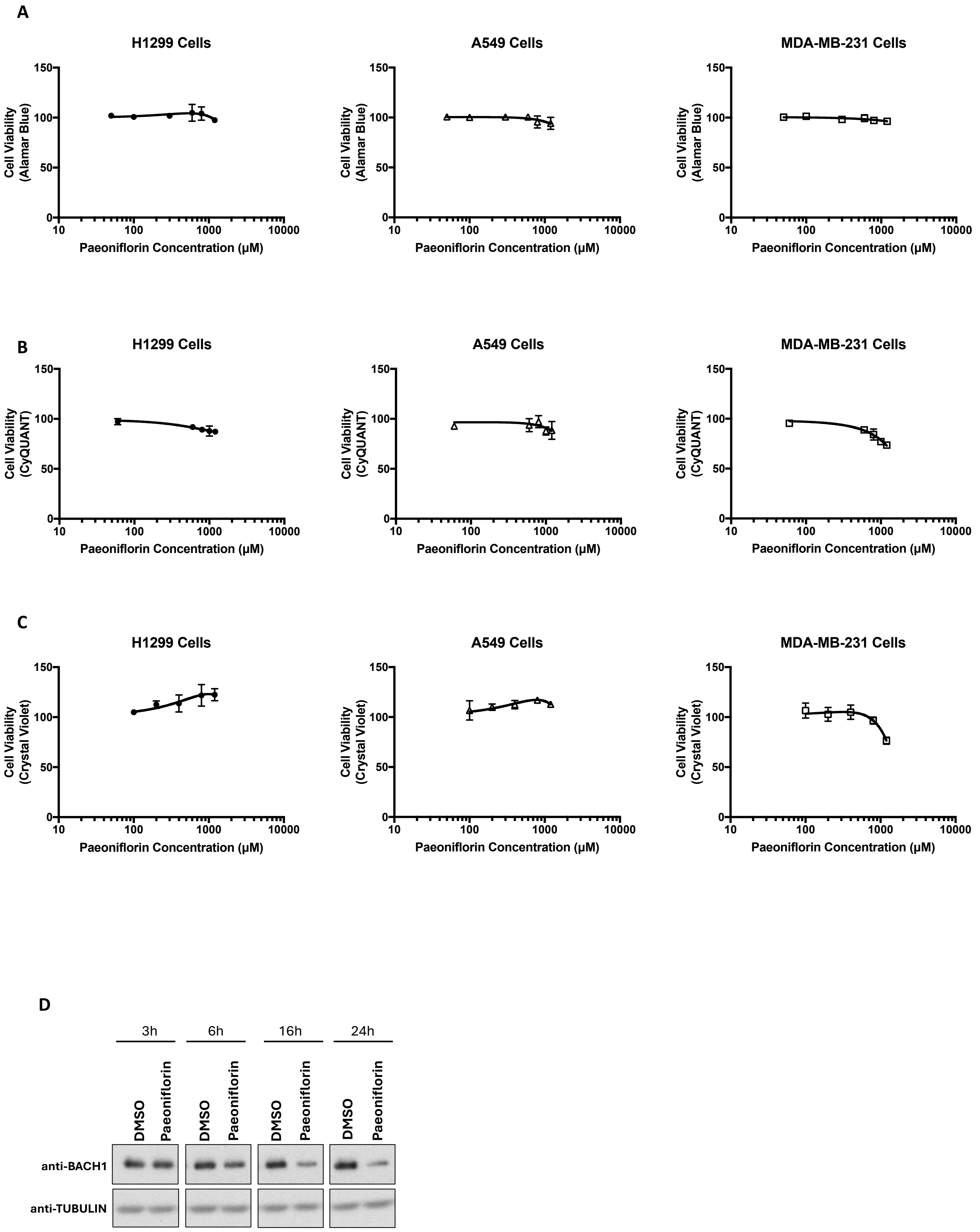
A
H1299 Cells
A549 Cells
MDA-MB-231 Cells
Cell Viability (Alamar Blue)
Paeoniflorin Concentration (µM)
B
H1299 Cells
A549 Cells
MDA-MB-231 Cells
Cell Viability (CyQUANT)
Paeoniflorin Concentration (µM)
C
H1299 Cells
A549 Cells
MDA-MB-231 Cells
Cell Viability (Crystal Violet)
Paeoniflorin Concentration (µM)
0
50
100
150
10
100
1000
10000
D
3h
6h
16h
24h
DMSO
Paeoniflorin
anti-BACH1
anti-TUBULIN

Suppl S4

A

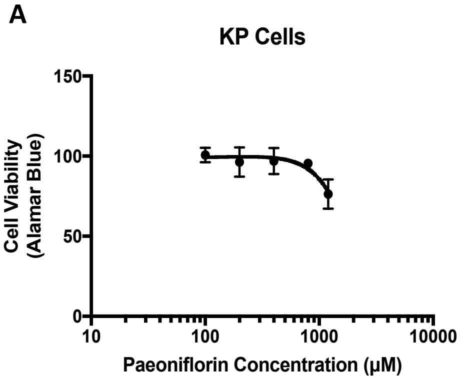
KP Cells
Cell Viability (Alamar Blue)
150
100
50
0
10
100
1000
10000
Paeoniflorin Concentration (μM)

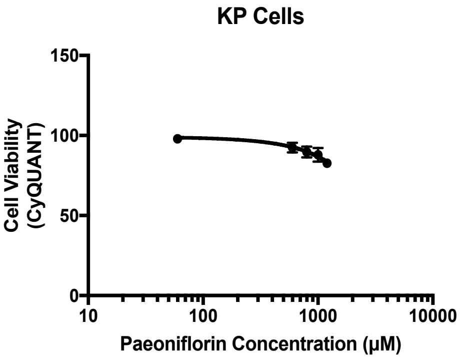
KP Cells
Cell Viability (CyQUANT)
150
100
50
0
10
100
1000
10000
Paeoniflorin Concentration (μM)

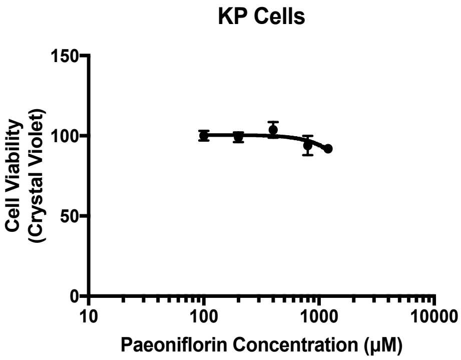
KP Cells
Cell Viability (Crystal Violet)
150
100
50
0
10
100
1000
10000
Paeoniflorin Concentration (μM)

B

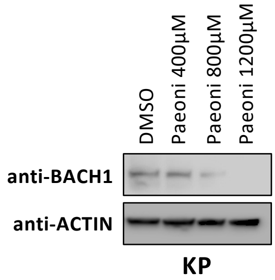
DMSO
Paeoni 400μM
Paeoni 800μM
Paeoni 1200μM
anti-BACH1
anti-ACTIN
KP

Suppl S5

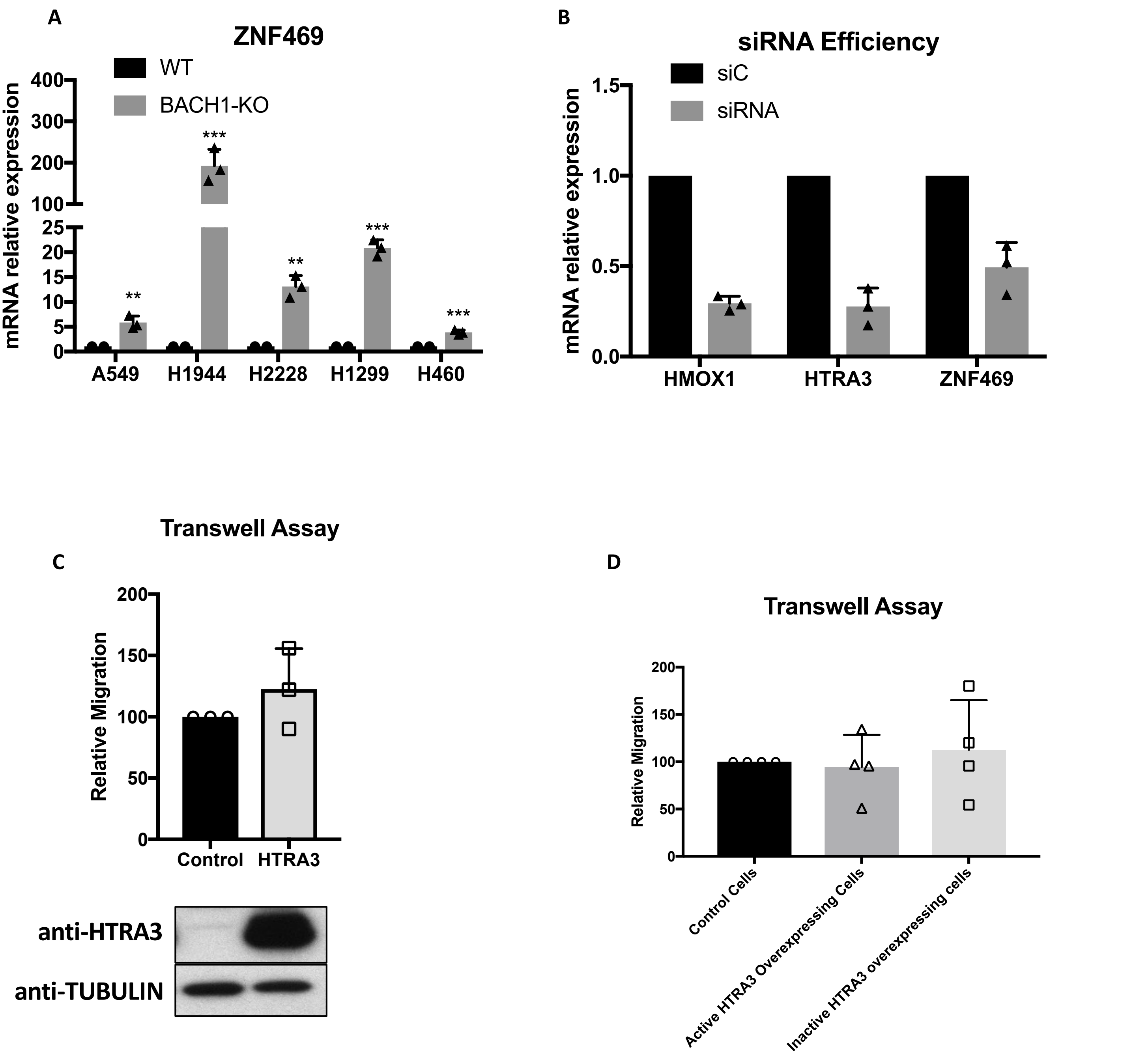

A
ZNF469
WT
BACH1-KO
mRNA relative expression
400
300
200
100
25
20
15
10
5
0
**
***
**
***
***
A549
H1944
H2228
H1299
H460
B
siRNA Efficiency
siC
siRNA
mRNA relative expression
1.5
1.0
0.5
0.0
HMOX1
HTRA3
ZNF469
Transwell Assay
C
200
150
100
50
0
Relative Migration
Control
HTRA3
anti-HTRA3
anti-TUBULIN
D
Transwell Assay
200
150
100
50
0
Relative Migration
Control Cells
Active HTRA3 Overexpressing Cells
Inactive HTRA3 overexpressing cells

Suppl S6
